# Supplementary material for: Realization of all-band-flat photonic lattices
Source: Nat Commun. 2024 Feb 19;15:1484. doi: 10.1038/s41467-024-45580-w (PMC10876559; doi:10.1038/s41467-024-45580-w)
Supplement: Supplementary file 1 — Supplementary Information [file 41467_2024_45580_MOESM1_ESM.pdf]

Supplementary Information: Realization of all-band-flat photonic lattices

Jing Yang[1,2,5], Yuanzhen Li[2,5], Yumeng Yang[2,5], Xinrong Xie[2,5], Zijian Zhang[2,5], Jiale Yuan[1], Han Cai[1,3], Da-Wei Wang[1,3,4,*], and Fei Gao[1,2,5,†]
[1]Zhejiang Province Key Laboratory of Quantum Technology and Device, School of Physics, and State Key Laboratory for Extreme Photonics and Instrumentation, Zhejiang University, Hangzhou 310027, China
[2]ZJU-Hangzhou Global Science and Technology Innovation Center, College of Information Science and Electronic Engineering, Zhejiang University, Hangzhou 310027, China
[3]College of Optical Science and Engineering, Zhejiang University, Hangzhou 310027, China
[4]CAS Center for Excellence in Topological Quantum Computation, University of Chinese Academy of Sciences, Beijing 100190, China
[5]International Joint Innovation Center, Key Laboratory of Advanced Micro/Nano Electronic Devices & The Electromagnetics Academy at Zhejiang University, Zhejiang University, Haining 314400, China
corresponding author: D.W.W. (dwwang@zju.edu.cn), F.G. (gaofeizju@zju.edu.cn)
(Dated: November 11, 2023)

CONTENTS

I. Eigenspectrum of the strained honeycomb lattice 2
  A. Three-mode JC model 2
  B. Next-nearest-neighbor coupling 2
  C. Imaginary coupling strength 3

II. Engineering of coupling strength 4

III. The reflection spectrum and the band flatness 5

IV. Mode distribution 5

V. Selective excitation with phase array source 7

VI. Applicability in three-dimensional lattices 8

VII. Nonlinearity in microwave resonators 8

VIII. Dynamic modulation of the coupling strength 9

IX. Nonlinear dynamics of ABF lattices 10

References 11

I. EIGENSPECTRUM OF THE STRAINED HONEYCOMB LATTICE

A. Three-mode JC model

Here we analytically derive the eigenspectrum in Eq. (2) of the main text. The Hamiltonian of the resonator lattices is

$$H = t_0[\sum_{i,j,k}(\sqrt{i}b^{\dagger}_{i-1jk} + \sqrt{j}b^{\dagger}_{ij-1k} + \sqrt{k}b^{\dagger}_{ijk-1})a_{ijk} + H.c.]. \tag{S1}$$

This tight-binding model can be mapped to the Fock-state lattices of three-mode JC model with the Hamiltonian

$$H_0 = t_0\sum_{n=1}^{3}(c_i\sigma^+ + \sigma^- c^{\dagger}_i) \tag{S2}$$

where t_0 is the vacuum Rabi frequency, c_i and $c^{\dagger}_i$ are the bosonic annihilation and creation operators of the three cavities, and $\sigma^+ = |b\rangle\langle a|$, $\sigma^- = |a\rangle\langle b|$ are the raising and lowering operators of a two-level atom, which has an excited state $|b\rangle$ and a ground state $|a\rangle$. The Fock state with i, j, k photons in three cavities and two-level atom in the state $|b/a\rangle$ is denoted as $|b/a, i, j, k\rangle_c$, which corresponds to B_{ijk} and A_{ijk} in our photonic lattice. The Hamiltonian H_0 conserves the total excitation number $N = i + j + k + (\xi + 1)/2 = 9$, where $\xi = 1$ for states $|b, i, j, k\rangle_c$ and $\xi = -1$ for states $|a, i, j, k\rangle_c$. The Eq. (S2) can be diagonalized by introducing three collective modes $d^{\dagger}_0 = \frac{1}{\sqrt{3}}\sum_{j=1}^{3} c^{\dagger}_j$ and $d^{\dagger}_{\pm} = \frac{1}{\sqrt{3}}\sum_{j=1}^{3} c^{\dagger}_j e^{\pm i2\pi/3j}$. Then Eq. (S2) is rewritten as a single-mode JC model Hamiltonian,

$$H_0 = \sqrt{3}t_0(d_0\sigma^+ + d^{\dagger}_0\sigma^-), \tag{S3}$$

whose eigenstates are $|\psi^{\pm}_{m,C}\rangle = (|b; m-1, m_+, m_-\rangle_d \pm |a; m, m_+, m_-\rangle_d)/\sqrt{2}$ for $m = 1,...,N$ with eigenenergies $E_{\pm m} = \pm\sqrt{3m}t_0$ and $|\psi_{0,C}\rangle = |\downarrow; 0, m_+, m_-\rangle_d$ with eigenenergy $E_0 = 0$. Here $m, m_{\pm}$ are the photon number in the $d_0, d_{\pm}$ collective modes, and $C = m_+ - m_-$ plays the role of the lattice momentum in an infinite lattice, determining the chirality of each eigenstate.

The explicit wave function is obtained by expanding $|\psi^{\pm}_{m,C}\rangle$ in the d_0 and $d_{\pm}$ modes as

$$|b/a; m, m_+, m_-\rangle_d = \frac{(d^{\dagger}_0)^m(d^{\dagger}_+)^{m_+}(d^{\dagger}_-)^{m-}}{\sqrt{m!m_+!m_-!}}|b/a; 0, 0, 0\rangle_c, \tag{S4}$$

and then replacing $d^{\dagger}_0, d^{\dagger}_{\pm}$ with $c^{\dagger}_i$. For example, the spatial distribution of the eigenstates with $m = 0$ are

$$\begin{aligned}|a; 0, m_+, N - m_+\rangle_d &= \frac{((d^{\dagger}_+)^{m_+}(d^{\dagger}_-)^{N-m+}}{\sqrt{m_+!(N - m_+)!}}|a; 0, 0, 0\rangle_c \\ &= \sum_{ijk} P^C_{ijk}|a; i, j, k\rangle\delta_{N,i+j+k},\end{aligned} \tag{S5}$$

where the coefficients read

$$P^C_{ijk} = \sum_{n_1n_2n_3}\frac{\sqrt{m_+!(N - m_+)!i!j!k!}e^{i\frac{2\pi}{3}(2n_1-2n_2-i+j)}}{\sqrt{3^N}n_1!n_2!n_3!(i - n_1)!(j - n2)!(k - n_3)!}\delta_{m_+,n_1+n_2+n_3}. \tag{S6}$$

P^C_{ijk} is also the amplitude of the eigenmode with chirality C on the site A_{ijk}. The field distribution of other eigenmodes can be analytically obtained in a similar way.

B. Next-nearest-neighbor coupling

The higher-order cavity modes around 12.5 GHz introduce the next-nearest-neighbor (NNN) coupling between resonators, the interaction Hamiltonian is

$$H_{NNN} = \kappa\sum_{i,j,k}[\sqrt{i(j + 1)}a^{\dagger}_{i-1j+1k}a_{ijk} + \sqrt{i'(j' + 1)}b^{\dagger}_{i'-1j'+1k'}b_{i'j'k'} + H.c.], \tag{S7}$$

with κ being the coupling strength. H_{NNN} can be mapped to the interaction Hamiltonian of the direct coupling between cavities

$$H_{NNN} = \kappa \sum_{n=1}^{3} (c_i^\dagger c_i + H.c.), \tag{S8}$$

which can be transformed to the d_0, $d_\pm$ modes as

$$H_{NNN} = \kappa(2d_0^\dagger d_0 - d_+^\dagger d_+ - d_-^\dagger d_-). \tag{S9}$$

The eigenspectrum of the total Hamiltonian $H_0 + H_{NNN}$ can be obtained by solving the 2×2 matrices in the basis $|b; m-1, m_+, m_-\rangle$ and $|a; m, m_+, m_-\rangle$ for $m = 1,...,N$,

$$\begin{pmatrix} \kappa(3m-1-N) & \sqrt{3m}t_0 \\ \sqrt{3m}t_0 & \kappa(3m-N) \end{pmatrix}, \tag{S10}$$

with the eigenenergy

$$E_{\pm m} = \kappa(3m - N - 1/2) \pm \frac{\sqrt{(\kappa^2 + 12mt_0^2)}}{2}. \tag{S11}$$

For $m = 0$, the NNN terms introduce an energy shift $E_0 = -N\kappa$.

C. Imaginary coupling strength

The fundamental mode of a single resonator is not a perfect TM mode. We simulate the spatial distribution of coupled modes of two resonators in Fig. S1. The components of the electric field in the x-y directions induce the propagating modes in coupling channels, thus resulting in weak indirect coupling between neighbouring resonators. Such indirect couplings are described by imaginary coupling strengths. We take the two coupled resonators as an example to explain the influence of imaginary coupling on the DOS $D(v)$.

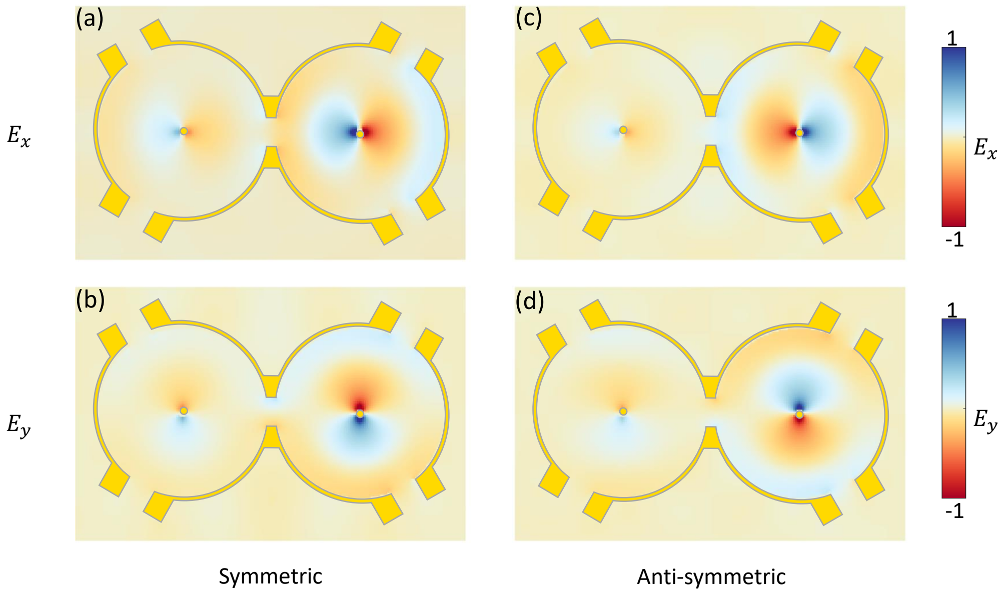

Figure S1. Field distribution of two coupled resonators. (a) and (b) The field distribution of the symmetric mode in the x-y plane. (c) and (d) The field distribution of the anti-symmetric mode in the x-y plane. The numerical simulation of two coupled resonators was conducted with CST Microwave Studio.

The dynamics of the field of two identical coupled resonators are described with the following set of coupled equations:

$$\begin{aligned} \frac{d}{dt}a_1 &= (i\omega_0 - \gamma)a_1 + i(t + it')a_2 + i\kappa F_1 e^{i\omega t} \\ \frac{d}{dt}a_2 &= (i\omega_0 - \gamma)a_2 + i(t^* + it')a_1. \end{aligned} \tag{S12}$$

$a_{1,2}$ are the field amplitude of two resonators, w_0 is the frequency of the fundamental mode in each resonator, t and t' are the real and imaginary parts of coupling strength. F_1 is the complex amplitude of the resonant excitation source at a_1, defined as $F_1 = 1$. Fig. S2 shows the spectra of $|a_1|^2$ with $t' = 0$ and -0.0004 GHz. When $t' = 0$, the two coupled modes have the same amplitude. When $t' = -0.0004$ GHz, the amplitude of the lower frequency mode is larger. The result is consistent with the asymmetry between the DOS $D(v)$ of the negative and positive Landau levels in the main text.

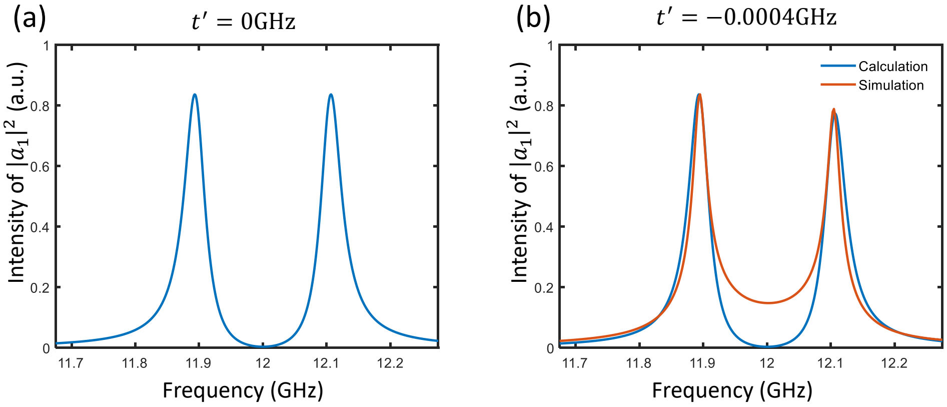

Figure S2. Spectra of two resonators with imaginary coupling strengths. (a) and (b) correspond to imaginary coupling strengths $t' = 0$ and -0.0004 GHz, respectively. Blue and red lines are calculated and simulated results, respectively.

II. ENGINEERING OF COUPLING STRENGTH

According to the tight binding approximation, the coupling factor is determined by the field overlapping integral [1]

$$t_0 = \int dr^3 C \cdot \mathbf{E}(\mathbf{r}_1) \cdot \mathbf{E}(\mathbf{r}_2), \tag{S13}$$

$\mathbf{E}(\mathbf{r}_1)$ and $\mathbf{E}(\mathbf{r}_2)$ represent the fields of neighboring resonators. C is the coefficient relating to the resonator geometry. To simplify analysis, we approximately consider the fields overlapping in the coupling channel. In the coupling channel, the fields $\mathbf{E}(\mathbf{r})$ manifest themself as waveguide modes. We take the $E_z(\mathbf{r})$ components, whose exact expression is

$$E_z(\mathbf{r}) = \sin(\pi x/d)e^{ik_y y}, \tag{S14}$$

where $k_y = \sqrt{k_0^2 - (\frac{\pi}{d})^2}$, and d represents the width of coupling waveguides. To satisfy the tight binding approximation, we ensure the couplings occur between evanescent fields, thus t_0 being real. According to Eq. $(S14)$, when k_y is pure imaginary, the field is totally evanescent. When the length of coupling channel is fixed, the magnitude of t_0 is controlled by the decay length $\delta = \frac{1}{ik_y}$ of evanescent fields. To realize the imaginary k_y, we adopt a small d such that the operational frequency w_0 is lower than the cutoff frequency w_{co} of coupling waveguide. Therefore, we can use the width d to tune the decay length, and thus tune the coupling strength. Fig. S3 shows the contrast overlap of evanescent fields by changing widths, while the waveguide lengths $l_c o = 2$ remain the same. The width of $d = 4$ results in a smaller overlap than that of $d = 8$ mm. According to Eq. $(S14)$, it concludes that the narrower waveguides lead to weaker couplings than the wider ones.

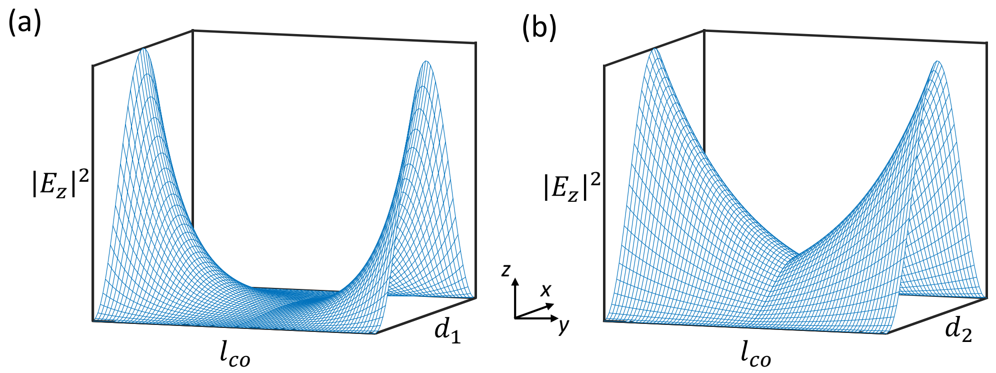

Figure S3. Intensity of $|E_z|^2$ in the coupling channel of two resonators for width $d_1 = 4$ mm (a) and $d_2 = 8$ mm (b). The coupling waveguide is along the y direction, with $l_{co} = 2$ mm. To visualize the field strength, the x and y axes have different scales.

III. THE REFLECTION SPECTRUM AND THE BAND FLATNESS

The experimental determination of the local DOS is obtained through the reflection coefficient on each site $R(\mathbf{r}_j, \nu)$, which is directly measured by a vector network analyzer. The reflection coefficient is,

$$R(\mathbf{r}_j, \nu) = 1 - i \sum_m \frac{|\Phi_m(\mathbf{r}_j)|^2}{\nu - \nu_m + i\gamma}. \tag{S15}$$

The reflection coefficient is connected to the intensity of the mode functions.

Fig.2 (b) of the main text shows the experimentally measured DOS of the lattices. The flatness of this band can be characterized by the ratio between the linewidth of each band γ_m and that of a single resonator γ. The ratio of each Landau level is listed in Tab. S1.

Table S1. The flatness of bands

m	-9	-8	-7	-6	-5	-4	-3	-2	-1	0	1	2	3	4	5	6	7	8	9
γ/γ_0	0.6	0.8	1	0.8	1	1	1.1	1.4	1.3	1.7	1.1	1.1	1.3	1.4	1.2	1	0.8	0.8	0.6

We notice that the widths of all the flatbands are approximately the same as that of a single resonator, which demonstrates high flatness of the bands. For some of the high pseudo-Landau levels, the width of a band is even smaller than that of a single resonator. This is because when we measure the bandwidth of a single resonator, all openings are open to the air, while when we measure that of the lattice, the openings are connected such that the loss to free space is reduced.

IV. MODE DISTRIBUTION

In Fig. S4, we show the experimentally measured mode distribution of all the flatbands. The eigenmodes in $m = 0$ are localized within the incircle of the triangular lattice boundary. The distribution moves towards the three corners for $m = 1$, 2, and 3. For $m = 4$, 5, and 6, the modes are mainly localized at edges. For $m = 7$, 8, and 9, the modes gradually shrinks to the lattice center.

In order to quantitatively characterize the variance of the mode, we calculate the standard deviation of field distribution,

$$\sigma_m = \frac{\sum_i \Phi_m(\mathbf{r}_i) \cdot \sqrt{\langle \mathbf{r}_i{}^2 \rangle - \langle \mathbf{r}_i \rangle^2}}{\sum_i \Phi_m(\mathbf{r}_i)}, \tag{S16}$$

where $\Phi_m(\mathbf{r}_i)$ is the mode intensity at location $\mathbf{r}_i$ for the mth Landau levels. Fig. S5 shows σ_m of two sublattices. We note that σ_m increases from $m = 0$ to ± 5 and then decreases, indicating that the modes spread from the center to the edges when m increases from 0 to $N/2$, and then shrink to the center when m increases from $N/2$ to N.

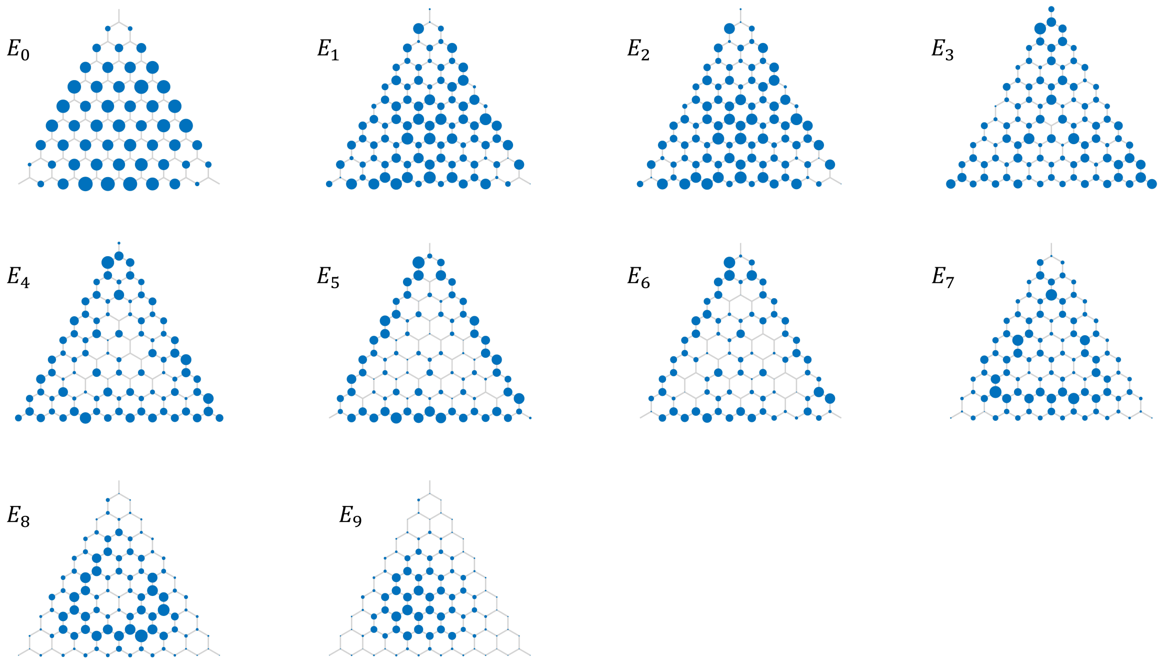

Figure S4. The measured mode intensities corresponding to the pseudo-Landau levels of indices $m = 0, 1, ..., 9$, respectively. The radii of the circles are proportional to local density of states $D(\mathbf{r}_j, \nu)$ on each site. The modes localized within the incircle of the triangular lattice boundary for $m = 0$, and move towards three corners for $m = 1$, 2, and 3. For $m = 4$, 5, and 6, the modes are mainly localized at three edges. For $m = 7$, 8, and 9, the modes gradually shrink to the lattice center.

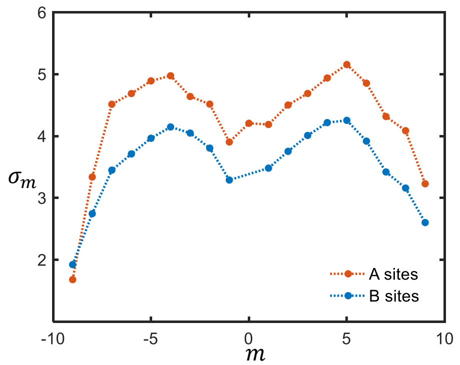

Figure S5. The standard deviation of field distribution σ_m for A (red) and B (blue) sublattices, which characterizes the variance of the mode distribution. σ_m increases from $m = 0$ to ± 5 and then decreases, indicating that the modes spread from the center to the edges when m increases from 0 to $N/2$, and then shrink to the center when m increases from $N/2$ to N.

We show the fidelity of the experimental results of the modes in Fig. S6 (in comparison with the theoretical calculation) for different bands, which is defined as

$$F(m) = \frac{\sum_i D_t(\mathbf{r}_i, \nu_m) \cdot D_e(\mathbf{r}_i, \nu_m)}{\sqrt{|\sum_i D_t^2(\mathbf{r}_i, \nu_m)| \cdot |\sum_i D_e^2(\mathbf{r}_i, \nu_m)|}}, \tag{S17}$$

where $D_t(\mathbf{r}_i, \nu_m)$ is the theoretical local DOS at the position $\mathbf{r}_i$ and eigenfrequency ν_m for the mth pseudo-Landau level and $D_e(\mathbf{r}_i, \nu_m)$ is the experimentally measured local DOS integrated in the frequency range $\nu_m \pm$ 5 MHz. Except for $m = \pm 9$, the fidelity of the flatbands is higher than 0.85.

We compared our current scheme of realizing flat bands with other works in Tab. S2.

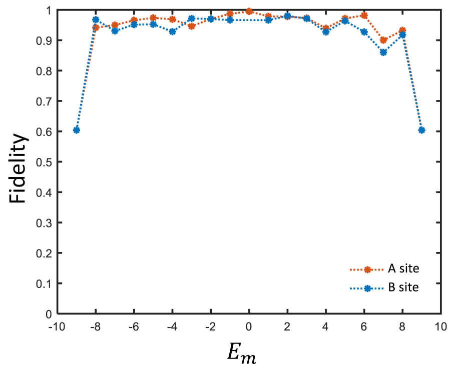

Figure S6. The fidelity of the experimental results for different flatbands. Red and blue lines correspond to A and B sites, respectively. Except for $m = \pm 9$, the fidelity of each band is higher than 0.85.

Table S2. Comparison between different flatband schemes

Article	Theory	Experimental realization	All-Bands flat	Edge effect
Our work	Bosonic statistical properties	Tuning coupling strength through the widths of connecting openings	Yes	No
[49] Phys. Rev. Lett. 121, 075502 (2018)	Synthetic magnetic field (AB caging)	Periodic time and space modulation of waveguide and incident wave	Yes	Yes
[53] Light Sci. Appl. 9, 146 (2020)	Synthetic magnetic field	Tuning coupling strength through the distance between dielectric cylinders	No	Yes
[54] Light Sci. Appl. 9, 144 (2020)	Synthetic magnetic field	Tuning coupling strength through the distance between micropillars	No	Yes
[57] Science 329, 544 (2010)	Synthetic magnetic field	Strain-induced pseudo–magnetic fields	No	Yes
[59] Nat. Photonics 7,153 (2013)	Synthetic magnetic field	Strain-induced pseudo–magnetic fields	No	Yes
[60] Nat. Phys. 15, 352 (2019)	Synthetic acoustic magnetic field	Contract acoustic Landau gauge potential by varying the shape of scatterers	No	Yes

V. SELECTIVE EXCITATION WITH PHASE ARRAY SOURCE

The phase array source is used to selectively excite the eigenmodes with different chiralities. Here we compare the results of $C = \pm 2$ for $m = 7$ in Fig. S7. When the phase differences between 3 sources are $\phi = -2\pi/3$, the eigenmode with $C = -2$ is excited, while for $\phi = 2\pi/3$, the eigenmode with $C = 2$ is excited. The two eigenmodes with opposite chiralities have the same field distribution.

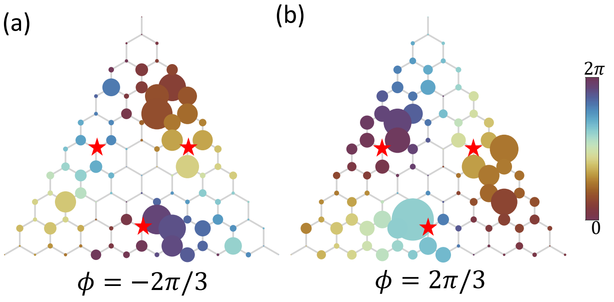

Figure S7. Excitation of eigenmodes with different chiralities for $m = 7$. The experimentally measured field distributions with excitation phases $\phi = -2\pi/3$ and $2\pi/3$ are shown in (a) and (b), corresponding to the eigenmodes with $C = -2$ and 2, respectively.

VI. APPLICABILITY IN THREE-DIMENSIONAL LATTICES

The JC model of four modes coupled to a two-level atom, is a three-dimensional (3D) FSLs of diamond geometry. The additional dimensionality could bring more intriguing physics, and such 3D FSLs also exhibit ABF energy spectra. However, it is challenging to construct these lattices with conventional dielectric waveguides or resonators, since their couplings in between are challenging to be engineered beyond the perturbative regime, especially in the 3D configuration. Our design is feasible to be extended into the 3D FSL. In each basic unit, each resonator couples with four neighboring resonators in a tetrahedron configuration rather than with three in a 2D plane. In the main text Fig.1, the field uniformly distributes along z axis. Such field distribution allows us to add extra openings on the resonator wall. This lays the foundation to design resonator lattices with 3D connectivity and tunable coupling strengths.
As a preliminary demonstration, we show a 3D lattices consists of 14 sites (Fig. S8(a)), which correspond to the 3D FSL with two excitations. Each B site (blue) is connected with four A sites (red). Fig. S8(b) shows the design of the 3D resonator array. We still choose the single-resonator mode which is uniform along the height of resonator. We elaboratedly design the coupling strengths with the basic one as $t_0 = 56$ MHz. Two adjacent resonators are shifted by 12 mm along z directions and connected by a coupling channel at 8 mm height. We tune the coupling strength by changing the width of coupling channel. Fig. S8(c) shows the simulated eigenspectrum of the resonator array, which shows five flatbands.

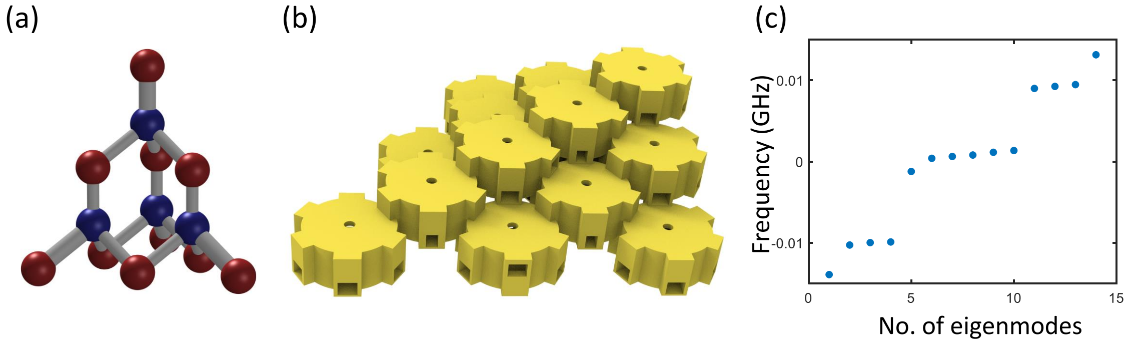

Figure S8. ABF three-dimensional diamond lattice. (a) Schematic of the 3D lattice. (b) Designed microwave resonators. (c) The simulated frequency spectra of the ABF diamond lattice.

VII. NONLINEARITY IN MICROWAVE RESONATORS

Microwave nonlinearity has been extensively realized with varactor diodes. Some examples are listed in Tab. S3. The basic principle of varactor nonlinearity can be found in the textbook [2]. A varactor works by locating it at and aligning with the maximum electric field components of microwave [3]. In our designed resonator, simulation results in Fig. S9(b) show that the maximum current is on the central metallic rod. We transform the conductive current into displacement current by cutting off the rod and introducing a tiny gap. Therefore, the maximum microwave current is transformed into maximum microwave electric field at the gap. We then bridge the tiny gap by welding the varactor (red part) on the rod, as shown in Fig. S9(a). Similar to those works in Tab. S3, the introduced varactors can lead to self-induced frequency shifts in our resonator as well, equivalent to on-site Kerr nonlinearity. More specifically, the 1SV309 varactor (TOSHIBA), whose operational frequency covers 3-30GHz, is suitable for our system.

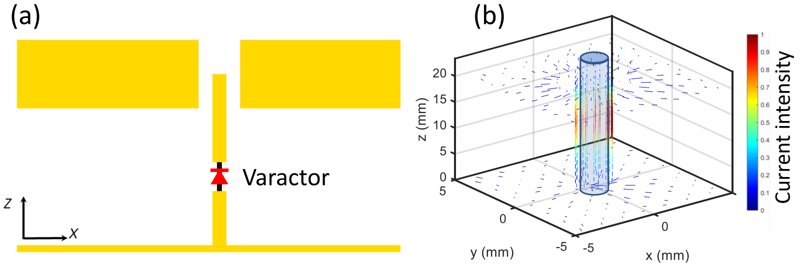

Figure S9. (a) Schematic of experimental realization of nonlinear Kerr effects in our structure. The rod in the middle of the resonator is connected by a varactor. (b) The current distribution on the metallic surface of a single resonator.

Table S3. Nonlinearity in microwave resonators

Article	System	Nonlinear element	Nonlinear type	Self-reduced frequency shift / coupling strength (ω_0)	Working frequency
[74]Phys. Rev. Lett. 121.163901 (2018)	resonators	varactor diodes	Kerr effect	0-0.06 / 0.03	1500 MHz
[75]Nat. Electron. 1, 178–182 (2018).	electronic circuit	nonlinear varactor diodes	Kerr effect	0.135-0.02 / 0.055	100 MHz
[80]Appl. Phys. Lett. 109, 111904 (2016)	mata-material	doubly resonant coupled split-ring resonator's (SRR)	Second-harmonic generation (SHG)	-	2000 MHz
[81]Nat. Commun. 10, 1102 (2019)	electronic circuit	nonlinear capacitor	Second-harmonic generation (SHG)	-	19 MHz

VIII. DYNAMIC MODULATION OF THE COUPLING STRENGTH

Using suitable microwave diodes, dynamic microwave metasurfaces have been widely achieved[4, 5]. With such scheme, we can also realize dynamic couplings by introducing diodes into the coupling channels. A diode along the z direction is introduced into and connected to inner wall of the coupling channel as shown in Fig. S10(a). Biased voltage can be used to switch the diode on and off. We numerically study a dimer system in Fig. S10(a) when the diode is on and off respectively. The simulated reflection spectra are shown in Fig. S10(b). The contrast spectra mean that the on and off states results in totally different coupling strength. Therefore, dynamically tuning the coupling configuration is feasible by embedding an electrically controlled diode in each coupling channel.

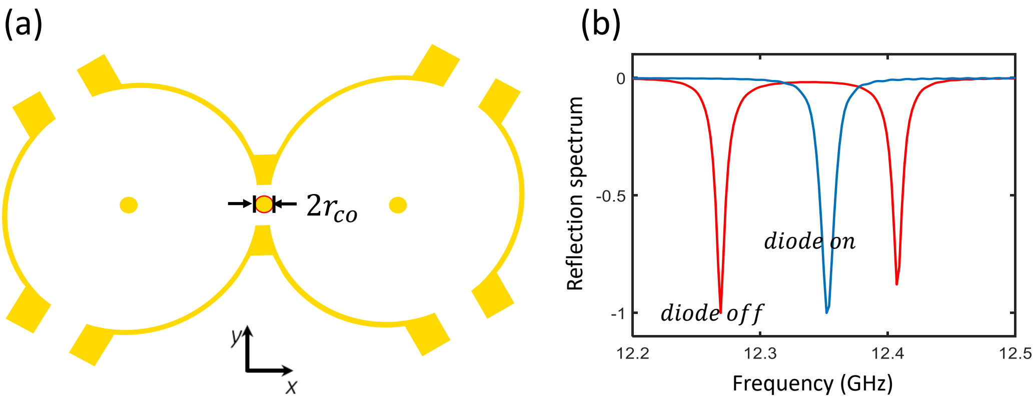

Figure S10. Schematic of experimental realization of coupling strength modulation in our structure. (a) We connect the top and bottom planes of the coupling channel by a metal rod with radius r_{co}. (b) The reflection spectrum when the diode is on (blue) and off (red).

IX. NONLINEAR DYNAMICS OF ABF LATTICES

Here we show the effect of Kerr nonlinearity on the ABF lattices. The evolution of the electric field amplitude ϕ_n in site n is governed by nonlinear dynamic equations,

$$-i\partial_t \phi_n = \sum_{n \neq m} t_{nm}\phi_m + g|\phi_n|^2\phi_n + i\gamma\phi_n, \tag{S18}$$

where t_{nm} are the coupling strengths between m and n sites, following the rules defined in the main text. g is the nonlinear susceptibility and γ is the dissipation of a single resonator. We set the minimal coupling strength $t_0 = 1$ and $\gamma = 0$ for simplicity. Without loss of generality, we choose the eigenstate with $C = N$ in the zeroth flatband as the initial state, which is a Gaussian wave packet localized at the center of the A-sublattice (Fig. S11(a)). Without nonlinearity, the state is static. An experimentally realizable nonlinearity can introduce an oscillation between different flatbands, demonstrating breathing dynamics as shown in Fig. S11(b). In particular, the initial state can evolve to B sites, and the dynamics are featured by quasi-periodic spreading and contraction, i.e., a breathing motion (see Fig. S11(d)). We make a fast Fourier transform (FFT) on the dynamics of the field amplitude and observe nonlinearity induced flatband splitting, as shown in Fig. S11(c) and Fig. S12.

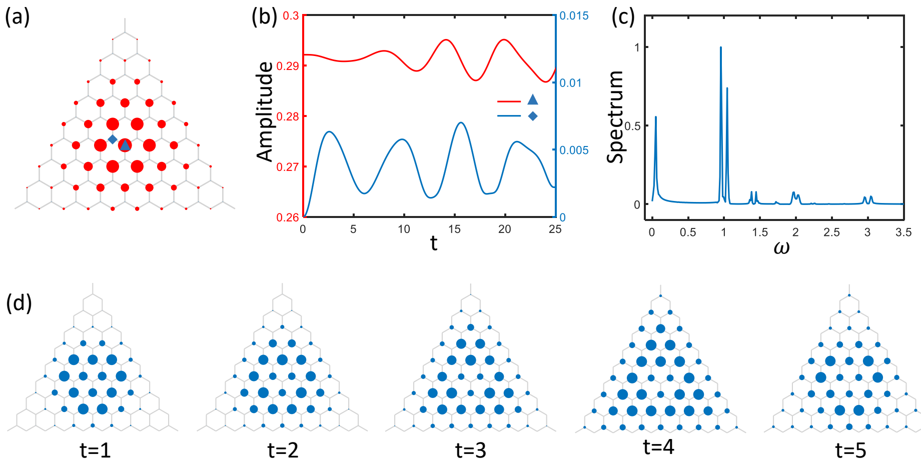

Figure S11. The dynamics of compact breathing mode of initial state $C = N$ in the zeroth flatband with nonlinear susceptibility $g = 1$. (a) The field distribution of the initial state. (b) The field amplitude on the sites marked by $\triangle$ and $\lozenge$ (A and B sublattices) in (a). Note that the scale of the field amplitude on each site is different from that in (a). (c) The FFT of the real part of the field on the $\lozenge$ site. (d) The field amplitude on B sites at time $t = 1, 2, 3, 4, 5$ during the breathing motion.

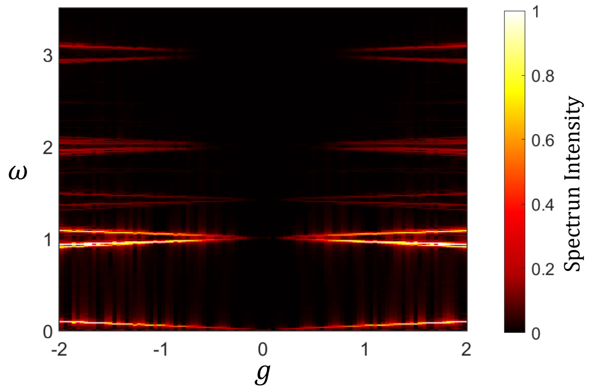

Figure S12. Frequency spectrum of breathing motion. The nonlinearity mixes the zeroth flatband with other flatbands, majorly $|m| = 1, 2, 4, 9$ bands. Higher nonlinearity results in larger mixing and larger flatband energy splittings.

[1] Yariv, A., Xu, Y., Lee, R. K. & Scherer, A. Coupled-resonator optical waveguide: a proposal and analysis. *Opt. Lett.* **24**, 711–713 (1999).
[2] Pozar, D. M. *Microwave engineering* (John wiley & sons, 2011).
[3] Wang, B., Zhou, J., Koschny, T. & Soukoulis, C. M. Nonlinear properties of split-ring resonators. *Opt. Express* **16**, 16058–16063 (2008).
[4] Li, L. *et al.* Electromagnetic reprogrammable coding-metasurface holograms. *Nat. Commun.* **8**, 197 (2017).
[5] Wu, G.-B. *et al.* A universal metasurface antenna to manipulate all fundamental characteristics of electromagnetic waves. *Nat. Commun.* **14**, 5155 (2023).
