# Supplementary material for: Realization of all-band-flat photonic lattices
Source: Nat Commun. 2024 Feb 19;15:1484. doi: 10.1038/s41467-024-45580-w (PMC10876559; doi:10.1038/s41467-024-45580-w)
Supplement: Supplementary file 3 — Editor Summary [file 41467_2024_45580_MOESM3_ESM.docx]

Here the authors experimentally realized a systematic approach to synthesize arbitrary-size two-dimensional all-band-flat photonic lattices, which pave a route for investigating flat-band related physics such as slow-light, nonlinear breathing, and dispersionless image transmission.
